# Structured approaches to improving outcomes of oral exam in medical and paramedical education: A systematic review and evidence-based framework

**DOI:** 10.1371/journal.pone.0355461

**Published:** 2026-08-10

**Authors:** Amir Torab-Miandoab, Mansour Ghafourifard, Sogand Habibi-Chenaran, Saeideh Ghaffarifar

**Affiliations:** 1 Medical Education Research Center, Health Management and Safety Promotion Research Institute, Tabriz University of Medical Sciences, Tabriz, Iran; 2 Department of Health Information Technology, School of Management and Medical Informatics, Tabriz University of Medical Sciences, Tabriz, Iran; Sri Ramachandra Institute of Higher Education and Research (Deemed to be University), INDIA

## Abstract

**Introduction:**

Oral examinations are central to medical and paramedical education for assessing reasoning, communication, and professional competencies, but remain challenged by issues of reliability, bias, and standardization. This systematic review synthesizes evidence on issues, requirements, and solutions to optimize oral exam outcomes in these fields.

**Materials and methods:**

Following PRISMA guidelines, a comprehensive search was conducted across 14 databases (e.g., PubMed, Scopus, Web of Science) up to June 23, 2025, using PICO-framed queries focused on medical and paramedical students. Inclusion criteria encompassed English-language full text studies on oral assessments; exclusions included editorials and reviews. Three independent reviewers screened titles, abstracts, and full texts, extracting data on study characteristics, challenges, requirements, solutions, and outcomes. Quality appraisal utilized QUADAS, with thematic analysis and matrix patterns to identify correlations.

**Results:**

From 25,594 records, 102 studies were included after deduplication and screening. The number of publications increased substantially after 2000, with the majority originating from the field of medicine (70.6%) and primarily addressing undergraduate (44.1%) and postgraduate (37.3%) education. Structured oral examinations (SOEs) dominated (56.9%), outperforming traditional formats in reliability (median Cronbach’s alpha 0.75; inter-rater interclass correlation coefficient (ICC) 0.47–0.82) and validity (modest correlations with objective structured clinical examinations (OSCEs), r=0.10–0.74). Key challenges included examiner variability (66.7%), student anxiety (41.2%), and logistical constraints (34.3%). The important requirements included assessment design, examiner calibration, technological integration, and equity. Solutions were emphasized on training (60.8%), standardization (56.9%), and technology (27.5%), yielding improved pass rates (50–100%) and satisfaction (72–96%). A comprehensive framework recommends multi-station objective structured viva examinations (OSVEs), hybrid delivery, combined rubrics, and artificial intelligence (AI)-assisted scoring for optimization. Limitations included generalizability (91.3%) and small samples (49.5%).

**Conclusions:**

This review outlines key challenges and strategies to improve the quality of oral examinations. Standardized oral examinations, supported by technology, offer fair and reliable assessments. The findings provide a framework for educators and researchers to optimize their use, while future studies should validate predictive validity and explore AI-driven solutions to reduce bias and enhance scalability.

## Introduction

An oral examination—also known as a viva or oral assessment—is a face-to-face evaluation method in which students verbally respond to examiners’ questions. It can be conducted one-on-one or in front of a panel and often involves discussion, case analysis, or problem-solving tasks [1]. The format allows examiners to probe the depth of understanding, clarify ambiguous responses, and assess how students organize and express their thoughts under pressure [2].

Oral exams are widely utilized across various disciplines and levels of education, particularly in high-stakes contexts [3]. In the medical and paramedical fields—including medicine, nursing, dentistry, physiotherapy, pharmacy, and public health—oral assessments play a critical role in evaluating students’ clinical decision-making, ethical reasoning, and professional judgment. They are also commonly used in interprofessional education settings, interdisciplinary case conferences, and competency-based assessments, reflecting their broad applicability and value in healthcare education [4].

The oral examination format offers several advantages over written and multiple-choice tests. It provides the opportunity for interactive assessment, immediate feedback, and tailored questioning based on student responses [5]. Oral exams closely mirror real-world clinical interactions, making them superior in assessing non-cognitive skills such as empathy, communication, professionalism, and the ability to think on one’s feet [6]. Its adaptability also allows examiners to explore complex reasoning processes, identify misconceptions, and evaluate critical thinking in ways that traditional written exams often fail to achieve [7].

Despite these strengths, oral exams have been persistently scrutinized for issues related to objectivity, reliability, inter-examiner variability, and susceptibility to bias. Concerns about consistency in questioning, examiner subjectivity, and limited standardization have raised questions about their fairness and validity, particularly when used in high-stakes settings [8]. Furthermore, the evolving complexity of medical education—with increased emphasis on competency-based assessment, digital innovation, and equity—has underscored the need for structured, evidence-informed improvements to oral examination methods [9].

Several educational frameworks and assessment models have been proposed to optimize oral exams, including structured oral examinations (SOEs), objective structured clinical examinations (OSCEs), and digital or video-recorded formats. However, the implementation of these strategies varies widely across institutions and regions, and the evidence regarding their effectiveness, feasibility, and educational impact remains scattered across the literature. A comprehensive understanding of the current challenges, best practices, and innovations is essential to inform educators, accreditation bodies, and policy-makers aiming to enhance the quality and outcomes of oral assessments [10].

In spite of their critical role in professional qualification and competency assurance, oral examinations in medical and paramedical sciences face persistent challenges related to standardization, validity, and educational effectiveness [11]. The lack of uniform criteria, examiner training, and robust quality assurance mechanisms has led to variability in outcomes and concerns about fairness and reliability. Moreover, emerging educational demands—such as competency-based learning, interprofessional collaboration, and technological integration—require updated and evidence-based approaches to oral assessment format [12].

While numerous interventions and modifications have been suggested in the literature to address these issues, there is a paucity of systematically synthesized evidence that comprehensively maps the existing challenges, stakeholder requirements, and viable solutions. Without such synthesis, efforts to reform oral examination practices risk being fragmented, inconsistent, or misaligned with educational goals. This systematic review aims to critically evaluate and integrate current research on the issues, requirements, and potential solutions to optimize oral examinations in the context of medical and paramedical education.

## Materials and methods

This systematic review was conducted in accordance with the Preferred Reporting Items for Systematic Reviews and Meta-Analyses (PRISMA) guidelines, which outline a standardized framework for reporting systematic reviews and meta-analyses [13]. This study was approved by the Ethics Committee of Tabriz University of Medical Sciences (Approval Code: IR.TBZMED.VCR.REC.1404.283).

### Review question

This systematic review was guided by the following PICO question:

Among medical and paramedical students undergoing oral examinations (Population), what assessment strategies, requirements or interventions (Intervention), compared to traditional methods or no interventions (Comparison), are effective in improving oral examination outcomes (Outcome)?

### Sources of information

A thorough literature search was performed across multiple electronic databases up to June 23 2025. These databases included PubMed, Web of Science, Scopus, CINAHL, ERIC, BIE, PsycINFO, IEEE, ProQuest, MEDLINE, Cochrane Library, Embase, SID, and ISC. In addition to peer-reviewed publications, we searched grey literature sources, including conference proceedings, books, dissertations, university repositories, institutional reports, and Google Scholar to reduce publication bias. These sources were identified through database searches, manual reference screening, and institutional repositories. Although grey literature was screened, only studies meeting all predefined eligibility criteria were included in the final review. To maintain awareness of new research, email alerts were configured in the databases to notify the authors of new studies matching the inclusion criteria, based on saved search queries as of June 23, 2025.

### Inclusion and exclusion criteria

Studies were eligible for inclusion if they focused on oral examinations within the context of medical and paramedical education. Only English-language articles with full text availability were considered. The editorials, commentaries, review papers, and opinion pieces were excluded from the review. There were no limitations on the year of publication.

### Search strategy

Search keywords were developed based on the study objectives, previously published literature, and input from subject-matter experts in medical informatics, health information management, and medical education. The detailed search syntax is outlined in Table 1. A medical librarian and an information specialist reviewed and approved the final search strategy. It is also noted that this search protocol was not pre-registered.

**Table 1:** The syntax of search strategy.

Databases	PubMed, Web of Science, Scopus, CINAHL, ERIC, BIE, PsycINFO, IEEE, ProQuest, MEDLINE, Cochrane Library, Embase, SID, ISC and Google Scholar
Date	up to 2025-6-23
Strategy	#1 AND #2
#1	(“Oral Exam” OR “Oral Examination” OR “Oral Assessment” OR “Oral Evaluation” OR “Oral Test” OR “Oral Appraisal” OR “Oral Quiz” OR “Oral Measurement” OR Viva OR Interview OR “Verbal Exam” OR “Verbal Examination” OR “Verbal Assessment” OR “Verbal Evaluation” OR “Verbal Test” OR “Verbal Appraisal” OR “Verbal Quiz” OR “Verbal Measurement” (
#2	(Medical OR Medicine OR Paramedical OR Paramedicine OR “Healthcare support” OR “Health Profession” OR “Health Occupation” OR “Health Technician” OR Health OR Paramedics OR “Clinical Support Service” OR “Allied Health” OR “Auxiliary Medical Service” OR Surgery OR radiology OR anesthesia OR emergency OR residents OR fellow OR anesthesiology OR specialist OR physiology OR anatomy OR psychiatry OR biochemistry OR pharmacology OR Laboratory OR Ophthalmology OR residency OR surgical OR Rehabilitation OR Physiotherapy OR Therapists OR pharmacy OR physician OR nursing OR Midwifery OR Dental OR Nutrition OR Dentistry OR therapy OR Optometry OR Audiometry OR clinical OR specialty OR clerkship OR internship)

Initially, potentially relevant articles were identified based on their titles and imported into EndNote. Duplicate entries were then removed. A more detailed screening followed, which involved reviewing the abstracts and full texts to further refine the list of eligible studies.

### Data collection procedure

Three researchers (A.T-M., S.Gh., and M.Gh.) independently evaluated the articles using a standardized spreadsheet. Each researcher assessed the titles for the inclusion and exclusion criteria, and articles that received two or more votes for exclusion were removed. In the next phase, the abstracts of the remaining studies were assessed independently using the same criteria, and those with at least two exclusion votes were discarded. Finally, the full texts were independently reviewed by all three authors, and studies flagged by at least two researchers were excluded.

A uniform data extraction form was employed to gather pertinent details from the selected articles. Information extracted included title, authors, year, country, study objective, study design, target population, educational setting, type of exam, technological component, challenges, requirements and needs, solutions, outcomes/key findings and limitation. All extracted data were consolidated and reviewed for accuracy by an independent investigator to ensure completeness and correctness. No data were missing from the final dataset.

### Quality evaluation

To support the inclusion and exclusion decisions, a formal quality appraisal was performed using the Quality Assessment of Diagnostic Accuracy Studies (QUADAS) checklist. This tool is designed to assess the methodological rigor and trustworthiness of diagnostic studies, either as a whole or by evaluating their validity and reliability separately [13].

The checklist comprises 14 questions, each scored as “yes,” “no,” or “unclear.” Two researchers independently assessed each study for potential sources of bias. A “yes” was assigned when the required information was clearly reported, “no” if the information was absent, and “unclear” if the report lacked sufficient detail. Based on existing guidelines, studies scoring 7 or more out of 14, with a majority of “yes” responses, were considered high quality, while those scoring below 7 were classified as low quality. Any disagreements between the reviewers were resolved through mutual discussion and consensus.

To visualize and report the findings, various software tools were used, including Microsoft Excel, XMind, an online word cloud generator, and the yED Graph Editor.

## Results

### PRISMA-based study selection

**Fig 1:**
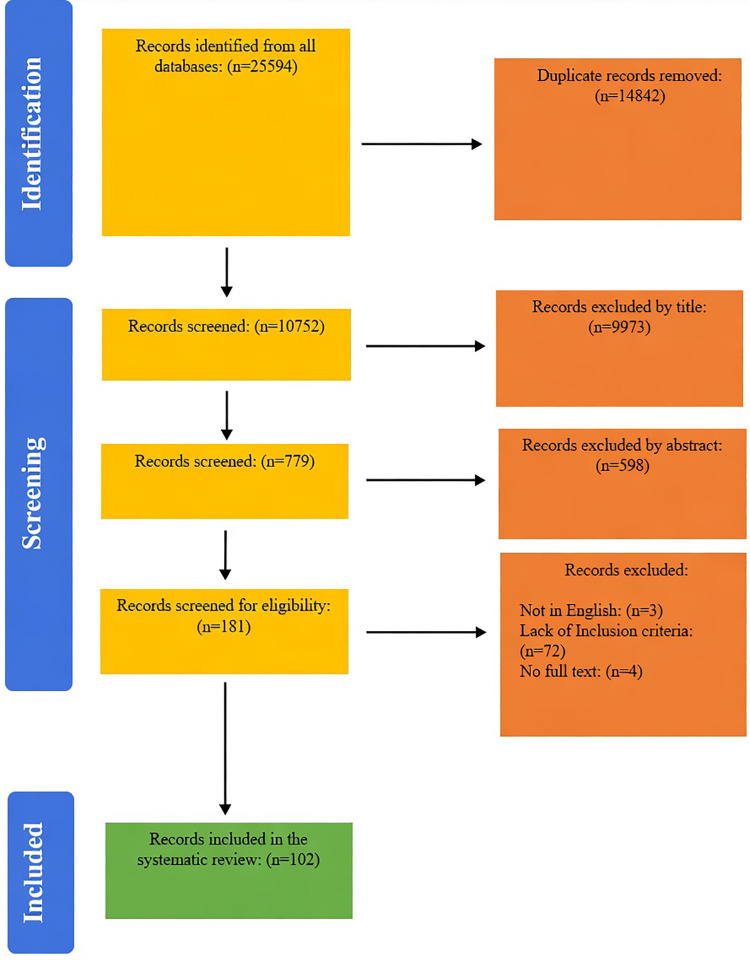
PRISMA flow diagram.

The comprehensive literature search across all databases identified 25,594 records. Following the removal of duplicates (n = 14,842), 10,752 unique records remained for initial title screening. Of these, 9,973 were excluded due to irrelevance based on title review, leaving 779 records for abstract screening. After evaluating abstracts, 598 records were excluded, resulting in 181 articles subjected to full text assessment for eligibility. Among these, 79 articles were excluded for the following reasons: non-English language (n = 3), failure to meet inclusion criteria (n = 72), and unavailability of the full text (n = 4). Ultimately, 102 studies fulfilled the eligibility criteria and were included in the systematic review. The study selection process is illustrated in the PRISMA flow diagram (Fig 1).

### Trends in scholarly output and global research distribution

**Fig 2:**
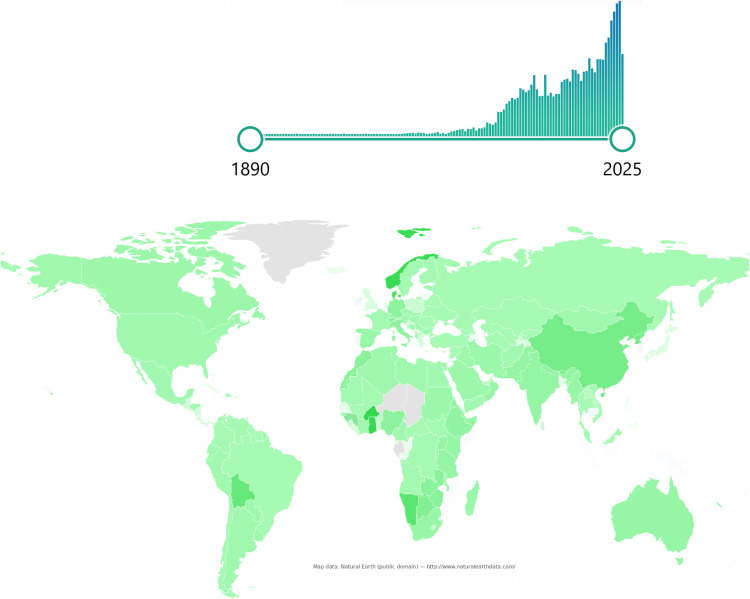
The trend of articles in the field of oral exam in medical and paramedical education in databases (The world map shown in Fig 2 was created by the authors using a public-domain base map (Natural Earth - http://www.naturalearthdata.com/)).

The histogram provided in Fig 2 illustrates the temporal distribution of publications on oral examinations in medical and paramedical education, based on data retrieved from multiple databases spanning 1890 to 2025. The findings indicate that research on oral examinations in medical and paramedical education was virtually absent prior to the mid-20th century, after which a gradual increase in scholarly output emerged. This growth accelerated markedly from the late 20th century onward, culminating in a peak in recent years, particularly approaching 2025. Early contributions during this period often focused on problem-solving formats within specific disciplines, such as family medicine, before evolving toward more diverse approaches in the late 20th and early 21st centuries. These later developments encompassed structured group examinations, as well as the integration of technological tools into viva voce assessments. Collectively, reviewing the trend reflects a sustained and expanding academic emphasis on oral assessment methods, driven by shifts in educational paradigms, advancements in assessment methodologies, and the growing recognition of oral examinations as a critical element of competency-based evaluation. Moreover, the accompanying world map depicts the geographical distribution of publications, with notable concentrations in North America, Europe, Oceania and selected regions of Asia, underscoring the global reach and collaborative nature of research in this domain (Fig 2).

### Keyword mapping and thematic patterns

**Fig 3:**
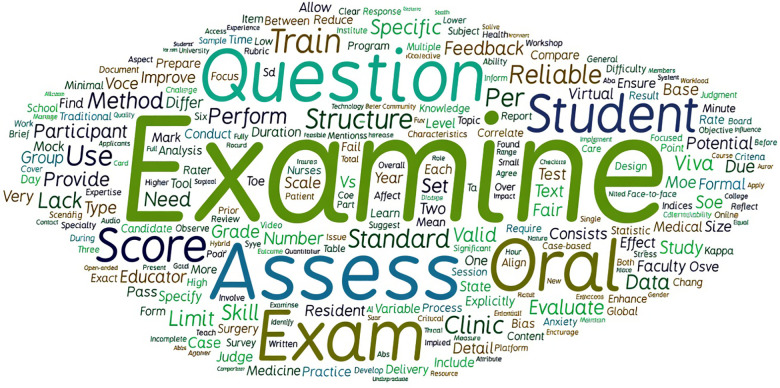
Frequent words cloud based on findings.

The word cloud, derived from a comprehensive analysis of literature on oral examinations in medical and paramedical education retrieved from multiple databases, visualizes the most frequently used terms in this research domain (Fig 3). Prominent keywords such as “Examine,” “Oral,” “Assess,” “Exam,” “Question,” “Student,” “Score,” “Valid,” and “Reliable” highlight core themes related to assessment methodologies, evaluation criteria, and the reliability of oral testing. Additional terms, including “Structure,” “Performance,” “Feedback,” and “Skill,” indicate an emphasis on structured assessment frameworks, performance appraisal, and the provision of constructive feedback. The presence of context-specific terms such as “Clinical,” “Medical,” “Case,” and “Viva” reflects the integration of oral examinations into clinical training and applied educational contexts. Collectively, this visualization underscores a sustained scholarly interest in the development and refinement of valid, reliable, and structured oral assessment tools tailored to the pedagogical requirements of medical and paramedical education.

### Overview of study designs

Analysis of the study designs reported in the 102 included articles revealed a diverse methodological landscape. Observational studies were the most common (n = 31), often incorporating inter-rater reliability assessments, generalizability analyses, and performance evaluations to examine real-world assessment practices. Experimental and quasi-experimental designs (n = 15) encompassed randomized controlled trials, crossover studies, and other interventional approaches, frequently aimed at comparing structured versus traditional examination formats and their effects on validity, reliability, and learner performance. Cross-sectional studies (n = 12) provided snapshot evaluations of perceptions, experiences, and scores, while qualitative studies (n = 10) explored thematic dimensions of stakeholder perspectives. Mixed-methods studies (n = 8) combined quantitative metrics with qualitative insights, offering a more comprehensive understanding of assessment processes. Additional designs included descriptive (n = 6), retrospective (n = 5), and comparative (n = 4) studies, which contributed foundational and historical perspectives. Reviews (n = 3), generalizability studies (n = 2), and other specialized approaches—such as pilot studies, survey-based investigations, and case studies (n = 6 collectively)—further underscored the methodological diversity supporting advancements in competency-based assessment within health professions education.

### Study populations and educational contexts

Among the included studies, the majority (n = 72; 70.6%) were conducted in the field of medicine, frequently within subspecialties such as surgery (n = 12), anesthesiology (n = 5), psychiatry (n = 4), pharmacology (n = 4), physiology (n = 4), and emergency medicine (n = 4), with additional representation from vascular surgery, internal medicine, and ophthalmology. Nursing was the second most represented discipline (n = 9; 8.8%), followed by dentistry (n = 3; 2.9%), physiotherapy (n = 2; 2.0%), and osteopathy (n = 2; 2.0%). Less frequently represented specialties included pharmacy, occupational therapy, radiology, and clinical psychology (each n = 1–2). This distribution highlights a predominant focus on medical disciplines, particularly those involving clinical and procedural training, where oral examinations serve as a critical component of competency assessment.

Educational levels spanned the full continuum of health professions training. Undergraduate learners constituted the largest cohort (n = 45; 44.1%), encompassing students in medicine (bachelor of medicine, bachelor of surgery)MBBS(or equivalent), nursing, dentistry, and physiotherapy at early to mid-program stages (first- to fourth-year). Postgraduate trainees—including residents, fellows, and certification candidates—were represented in 38 studies (37.3%), with particular emphasis on residency training (post-graduate year (PGY) 1–5) in specialties such as surgery and anesthesiology. Faculty members and practicing professionals were the target population in 12 studies (11.8%), often in the context of certification, recertification, or examiner training. The remaining studies (n = 7; 6.9%) involved mixed or unspecified levels, including pre-registration interns and community health workers.

Sample sizes ranged from small pilot studies (9–20 participants) to large multi-year cohorts exceeding 1,000 candidates, with a median of approximately 80 participants per study (interquartile range: 30–150). Where reported, participant numbers were often stratified by subgroups—such as residents by year (e.g., 36 first-year vs. 32 second-year trainees) or examination format (e.g., 63 in structured oral examinations vs. 60 in conventional formats). Reported response rates averaged 70–80%, although some studies noted attrition (e.g., 42 dropouts from 150 enrolled). Gender distribution, inconsistently documented, showed a female predominance in nursing and allied health cohorts (e.g., 42 females vs. 13 males in one physiology study).

Studies were conducted across diverse institutional settings, most commonly university-affiliated medical schools and teaching hospitals (n = 78; 76.5%), including institutions such as the University of Michigan, Harvard Medical School affiliates, and the All-India Institute of Medical Sciences. Multi-center collaborations were frequent (n = 18; 17.6%), involving networks such as the Southern Association for Vascular Surgery (USA) and international partnerships (e.g., University Sains Malaysia with the University of Otago, New Zealand). Professional board and certification contexts—such as the American Board of Emergency Medicine and the Royal College of Physicians and Surgeons of Canada—featured in six studies (5.9%).

### Oral examination formats and technological integration

Examinations were categorized by structural characteristics. Structured oral examinations were the most prevalent (n = 58; 56.9%), typically incorporating predefined questions, standardized scenarios, scoring rubrics, or checklists to enhance objectivity and reliability. Many of these formats were modeled on certification board practices (e.g., American Board of Surgery; Royal College of Physicians and Surgeons of Canada) and included variants such as mock oral examinations (n = 18; 17.6%) designed for preparatory purposes. Traditional, unstructured oral examinations accounted for 24 cases (23.5%) and were characterized by examiner-led, open-ended questioning, with greater subjectivity and variability in delivery. Virtual or online formats were reported in 12 cases (11.8%), reflecting adaptations to remote assessment—particularly in the post–COVID-19 period—while hybrid approaches appeared in 5 cases (4.9%). Less common formats included dialogic group orals (n = 1; 1.0%) and immersive simulations (n = 1; 1.0%). Three cases (2.9%) were not explicitly classified as examinations but evaluated curricula or surveys that incorporated oral assessment elements (Table 2).

**Table 2:** Distribution of oral exam types (N = 102) and technological components employed (N = 40 Cases with Technology).

Items		Number of Cases (%)
**Exam Type**	Structured	58 (56.9%)
	Traditional	24 (23.5%)
	Mock	18 (17.6%)
	Virtual/Online	12 (11.8%)
	Hybrid	5 (4.9%)
	Other (e.g., Dialogic, Immersive)	5 (4.9%)
**Technology Type**	Videotaping/Recording	18 (45.0%)
	Virtual Platforms (e.g., Zoom, Teams)	12 (30.0%)
	Digital Forms/Surveys (e.g., Google Forms)	9 (22.5%)
	Simulation/Imaging Software	6 (15.0%)
	Audio Recording Apps	2 (5.0%)
	Other (e.g., Electronic Checklists)	5 (12.5%)

Exam characteristics varied in duration (7 minutes to 4 hours, where specified) and delivery method, with face-to-face formats predominating (n = 78; 76.5%), followed by virtual (n = 12; 11.8%) and hybrid (n = 5; 4.9%) modalities. Question types were primarily case-based or clinical (n = 85; 83.3%), with open-ended formats in 42 cases (41.2%). Scoring methods in structured examinations favored rubric-based or global rating scales (e.g., Likert-type or percentage-based), whereas traditional formats relied predominantly on subjective consensus grading.

Overall, technological integration was limited, with 62 cases (60.8%) reporting no technology use and relying exclusively on in-person delivery with paper-based tools (e.g., printed checklists or question cards). Among the 40 cases (39.2%) that incorporated technology, the most common application was video recording (n = 18; 17.6%), used for independent rating, bias mitigation, and post-examination review (e.g., inter-rater reliability analysis). Virtual platforms such as Zoom, Microsoft Teams, or Google Meet were employed in 12 cases (11.8%), primarily for remote mock orals. Digital tools for feedback and data collection (e.g., Google Forms, Microsoft Forms, REDCap) were used in 9 cases (8.8%). Additional innovations included simulation software (Second Life; n = 1), audio recording applications (Audacity or Soundnote; n = 2), and image or presentation software (e.g., PowerPoint, OsiriX) for case display (n = 5). In structured examinations, technology frequently supported objectivity through encrypted question banks or electronic checklists, whereas adoption in traditional formats was limited (4 of 24 cases; 16.7%) (Table 2).

Examiners were generally multidisciplinary faculty or specialists (e.g., surgeons, psychiatrists, nurses), with numbers per examination ranging from 1 to 140. Examiner training was reported in 28 cases (27.5%), typically involving orientation or standardization sessions; in contrast, many unstructured formats reported the use of untrained examiners, contributing to procedural variability. Inter-rater reliability, assessed using measures such as Cohen’s kappa or the intraclass correlation coefficient (ICC), was reported in 15 cases (14.7%), reflecting ongoing efforts to reduce subjectivity.

### Challenges and proposed solutions in oral examinations

The identified challenges were systematically classified into six overarching domains through thematic coding: (1) examiner-related variability (e.g., subjectivity, bias, inconsistency), (2) student-related factors (e.g., stress, anxiety, preparedness), (3) logistical and resource constraints (e.g., time limitations, faculty availability), (4) reliability and validity concerns (e.g., low inter-rater agreement, limited generalizability), (5) format-specific limitations (e.g., subjectivity in traditional examinations, technical failures in virtual formats), and (6) equity-related issues (e.g., gender or racial bias, language barriers).

Examiner variability emerged as the most frequently cited challenge, reported in 68 studies (66.7%), reflecting persistent concerns over inconsistent scoring and halo effects. Student stress and anxiety were documented in 42 studies (41.2%), particularly in high-stakes or unstructured assessment settings. Logistical challenges were identified in 35 studies (34.3%), including time pressures and limited resources. Reliability and validity concerns were highlighted in 52 studies (51.0%), often attributed to small sample sizes or case-specific variance. Format-specific challenges, such as technical disruptions in virtual examinations, were reported in 18 studies (17.6%), while equity-related concerns were noted in 15 studies (14.7%).

These issues were more prevalent in traditional or unstructured examinations (28 studies; 27.5% of total) compared to structured formats, in which problems such as content overload or inflexible time limits were reported but were generally less severe. The shift toward virtual examinations—often accelerated by the COVID-19 pandemic (12 studies; 11.8%)—introduced challenges such as unstable internet connectivity but also improved accessibility for certain cohorts.

Proposed solutions were proactive and multifaceted, grouped into the following categories: examiner training and calibration, question and scoring standardization, use of multiple examiners, integration of technological tools, implementation of feedback mechanisms, and structural or format adjustments. Examiner training was the most frequently recommended intervention (62 studies; 60.8%), typically involving orientations, workshops, or peer review sessions to mitigate bias and improve scoring consistency. Standardization of questions and scoring—through structured checklists, rubrics, or predefined case scenarios—was suggested in 58 studies (56.9%), with the aim of enhancing reliability, often achieving inter-rater reliability coefficients exceeding 0.80 in multi-case assessments. The use of multiple examiners was advocated in 45 studies (44.1%), frequently employing consensus grading or independent scoring to reduce individual variability.

Technological interventions, such as video recording for post-hoc review or the use of secure online platforms, were reported in 28 studies (27.5%), particularly for addressing bias and enabling remote assessment. Feedback mechanisms, including post-examination debriefings and self-assessment opportunities, were noted in 32 studies (31.4%) to support learner development and promote iterative improvements in examination processes. Structural modifications—such as increasing the number of cases or adopting hybrid examination formats—were proposed in 40 studies (39.2%) to address logistical and equity-related challenges.

**Fig 4:**
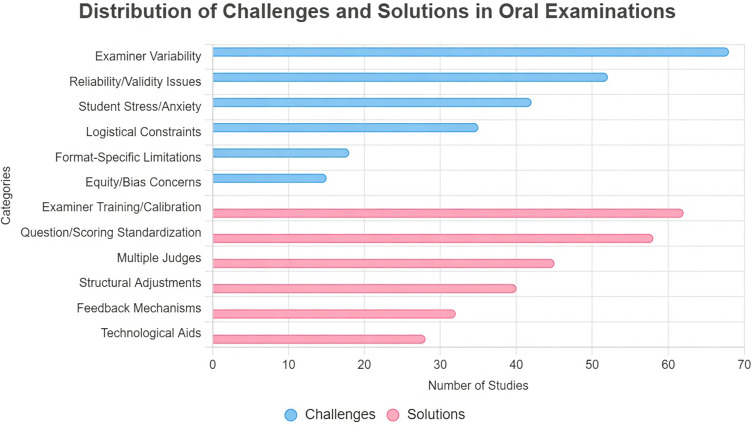
Distribution of challenges and solution in oral exam.

Solutions were frequently tailored to the specific challenge domain: for example, examiner training and scoring standardization addressed examiner variability in 85% of relevant studies, whereas technological interventions mitigated virtual examination challenges in 90% of such cases. In 15 studies (14.7%), pilot testing and validation procedures were implemented to ensure feasibility and optimize assessment design. Overall, structured and mock examination formats demonstrated superior effectiveness in addressing identified challenges, with reported improvements in inter-rater reliability (kappa coefficients >0.70) and enhanced learner satisfaction. The challenges and solutions are summarized in Fig 4.

### Requirements and needs for effective oral examinations

The analysis of extracted literature revealed a comprehensive set of requirements and needs essential for the effective design, implementation, and evaluation of oral examinations in medical and paramedical education. These requirements were categorized into several thematic domains (Table 3):

**Table 3:** Thematic categorization of requirements and needs for oral exam in medical and a paramedical education.

Domain	Subcategories	Representative Requirements
**Assessment Design & Structure**	- Structured formats - Blueprinting - Case diversity - Scoring rubrics	- Use of SOE - Standardized question formats - Multiple cases and examiners - Validated scoring criteria
**Examiner Preparation & Calibration**	- Training programs - Bias mitigation - Consensus building	- Examiner training to reduce variability - Double-blind procedures - Use of rating anchors - Peer observation and mentorship
**Candidate Experience & Support**	- Anxiety reduction - Feedback mechanisms - Preparation resources	- Mock oral sessions - Clear exam expectations - Formative feedback - Structured preparation strategies
**Technological Integration**	- Virtual platforms - App-based tools - Security protocols	- Stable online platforms (e.g., Zoom) - Audio/video recording for feedback - Secure proctoring methods - App compatibility and UI design
**Curricular Alignment & Validity**	- Competency frameworks - Cognitive domain coverage - Psychometric validation	- Alignment with CanMEDS/ACGME roles - Assessment of clinical reasoning - Pilot testing for validity - Structured question grids
**Operational & Logistical Considerations**	- Faculty coordination - Exam scheduling - Infrastructure needs	- Adequate faculty and examiners - Time allocation per question - Standardized documentation - multi-institutional collaboration

1. Assessment Design and Structure: A strong emphasis was placed on the use of SOEs to enhance fairness, reliability, and standardization. Studies consistently advocated for standardized question formats, blueprinted content, and validated scoring rubrics to ensure alignment with curricular objectives and clinical competencies. The inclusion of multiple cases and examiners was recommended to reduce variability and improve psychometric robustness.
2. Examiner Preparation and Calibration: Effective oral assessment was found to depend heavily on examiner training, including calibration sessions, mentorship programs, and consensus-building activities. Mechanisms to mitigate examiner bias—such as double-blind procedures, standardized rating anchors, and demographic balancing—were frequently cited. The need for ongoing professional development and feedback loops for examiners was highlighted to maintain consistency and improve scoring accuracy.
3. Candidate Experience and Support: Numerous studies underscored the importance of reducing student anxiety through clear communication of exam expectations, mock oral sessions, and supportive environments. Structured feedback mechanisms—both formative and summative—were deemed critical for enhancing learning outcomes and guiding remediation. Recommendations included adequate time allocation, transparent scoring criteria, and flexibility in question delivery to accommodate diverse learner needs.
4. Technological Integration: While traditional face-to-face formats remained dominant, there was growing interest in virtual oral examinations, particularly in response to logistical and accessibility challenges. Requirements for stable platforms, secure proctoring, and user-friendly interfaces were frequently mentioned, alongside the need for digital tools to support feedback and scoring. App-based assessments and video-enhanced formats were explored as innovative approaches to simulate clinical scenarios and enhance realism.
5. Curricular Alignment and Validity: Oral examinations were increasingly expected to assess higher-order cognitive skills, including clinical reasoning, decision-making, and communication. Alignment with competency-based frameworks (e.g., CanMEDS, ACGME) was emphasized to ensure relevance and educational impact. The need for content validity, construct validity, and predictive validity was recurrent, with several studies proposing pilot testing and psychometric analysis to support exam refinement.
6. Operational and Logistical Considerations: Successful implementation required adequate faculty resources, coordinated scheduling, and appropriate examination settings. Recommendations included standardized documentation, recording protocols, and quality assurance processes to support transparency and reproducibility. Multi-institutional collaboration and centralized oversight were proposed to enhance consistency across programs.

### Outcomes of oral examinations

Reliability emerged as a central concern, with structured formats consistently outperforming traditional ones. Cronbach’s alpha values ranged from 0.52 to 0.99 across studies, with a median of 0.75 indicating moderate to high internal consistency for SOEs. Inter-rater reliability, measured via ICCs or Cohen’s kappa, was generally moderate (kappa = 0.47–0.82), improving post-standardization or examiner training (from 0.49 to 0.82). Generalizability coefficients suggested that 6–10 cases or examiners were needed for reliability ≥0.80. Variability in examiner severity contributed significantly to score variance (84.1%), underscoring the need for calibration. Practice effects enhanced performance over repeated assessments (pass rates increasing from 75% to 100%), but halo effects were noted in candidate-specific rating.

Content and construct validity were supported in most studies, with SOEs demonstrating strong alignment with clinical competencies. Correlations between oral scores and written exams or OSCEs were modest (r = 0.10–0.74), indicating oral exams assess distinct skills like clinical reasoning and communication. Criterion validity was evident in associations with postgraduate performance (r = 0.37–0.45 with prior certification scores) and reduced racial grading disparities post-standardization. However, some studies reported low predictive validity for board certification pass rates (no significant correlation).

Pass rates varied widely (50–100%), with structured formats yielding higher consistency (77–100%). Mean scores ranged from 56.53% to 95.3%, often higher in hybrid or virtual models (91.98% in NCLEX/oral groups). Significant improvements were observed with training levels (PGY-3: 50% vs. PGY-5: 92%, p = 0.006) and interventions like mock orals (89% vs. national 86%). Gender and ethnic differences were noted, with females occasionally underperforming due to bias, though mitigated in structured formats.

Student satisfaction was high for structured and virtual formats (72–96% agreement on fairness and reduced stress), with preferences for SOEs over traditional viva (64–90%). Qualitative themes included enhanced reflection, reduced anxiety, and better alignment with real-world practice. Examiners valued SOEs for objectivity (83–100% agreement) but noted workload burdens (NASA TLX score: 59.6 ± 14.1). Feasibility was affirmed in resource-constrained settings, though time requirements (25–37.5 hours for 150 students) posed challenges.

Compared to written exams, orals better assessed higher-order skills (problem-solving, r = 0.80) but showed lower reliability without structure. Virtual formats were comparable to in-person (no significant differences in scores or pass rates, p > 0.05), with benefits in accessibility and cost (76–97% agreement). Feasibility metrics highlighted minimal costs ($34.60 per resident) but emphasized examiner training needs.

### Limitations of included studies

The most dominant limitation was restricted generalizability, appearing in 94 studies (91.3%). This was frequently attributed to single-institution or single-center designs, discipline-specific foci (e.g., psychiatry, pharmacology, or vascular surgery), or contextual factors such as regional or cultural settings, which limit extrapolation to broader educational or clinical environments. Small sample sizes emerged as the second most prevalent issue, cited in 51 studies (49.5%), often involving cohorts of fewer than 50 participants (e.g., 20–30 students or residents), thereby compromising statistical power, representativeness, and the ability to detect meaningful effects.

Potential biases were reported in 61 studies (59.2%), encompassing various forms such as selection bias (e.g., convenience or volunteer sampling), response bias in surveys, examiner bias due to familiarity or subjectivity, and reporting bias from self-administered questionnaires. This theme underscores inherent challenges in oral examination research, where interpersonal dynamics and non-randomized designs amplify subjectivity. Lack of quantitative data or rigorous statistical analysis was evident in 22 studies (21.4%), with many relying on qualitative insights, descriptive statistics, or unvalidated instruments (moderate Cronbach’s alpha values), which hinders objective evaluation of outcomes like reliability or validity.

Additional recurring limitations included a narrow scope or focus on specific topics/subjects (21.4%), such as single disciplines or exam formats, potentially overlooking interdisciplinary applications; insufficient details on examiner training or calibration (19.4%), which could affect scoring consistency and inter-rater reliability; and dependence on subjective measures (e.g., self-reported perceptions or surveys; 15.5%), introducing variability and potential inaccuracies. Absence of long-term follow-up or prospective data was noted in 13 studies (12.6%), limiting insights into sustained impacts on learning or clinical performance. Less frequent but notable issues encompassed incomplete or truncated data (8.7%), low survey response rates (6.8%), technical challenges (e.g., connectivity in virtual formats; 5.8%), retrospective designs (4.9%), and quasi-experimental approaches (1.9%), all of which pose risks to causal inference and reproducibility (see Table 4 for a comprehensive summary of these themes). All details of the articles included in the study are provided in S1 File.

**Table 4:** Summary of recurrent limitations Identified in the included studies.

Theme	Number of Studies	Percentage (%)
Limited generalizability	94	91.3
Potential bias	61	59.2
Small sample size	51	49.5
Lack of quantitative data/statistical analysis	22	21.4
Limited scope/focus	22	21.4
Lack of examiner training	20	19.4
Subjective measures	16	15.5
No long-term follow-up	13	12.6
Single institution/center	9	8.7
Incomplete data	9	8.7
Low response rate	7	6.8
Technical challenges	6	5.8
Retrospective design	5	4.9
Quasi-experimental design	2	1.9

### Patterns and associations across study variables

Based on the study dataset, we conducted a qualitative thematic analysis to identify patterns, correlations, and potential associations among the extracted variables. Table 5 presents the identified patterns across variable pairs.

**Table 5:** Matrix of observed patterns between study variables.

Row	Variable Pair	Observed Pattern	Meaningful Interpretation
1	Year × Country	Pre-2000: Mostly Canada/US (~1985); Post-2010: Diverse (Saudi Arabia, India, Brazil)	Shift from Western dominance to global representation
2	Year × Study Design	Pre-2000: Descriptive/quasi-experimental; Post-2010: Mixed-method/prospective	Transition to more rigorous and interventional designs
3	Year × Target Population	Pre-2000: Undergrad/preclinical; Post-2010: Postgrad/residents/elderly	Focus shift toward advanced and community learners
4	Year × Educational Setting	Pre-2000: Single university; Post-2010: Multi-institution/community	Broader and more diverse educational settings
5	Year × Type of Exam	Pre-2000: Structured/group; Post-2010: Virtual/app-based	Movement from in-person to hybrid/online formats
6	Year × Technological Component	Pre-2000: None; Post-2010: Video/apps/virtual (~0% vs. ~ 50%)	Strong increase in technology use
7	Year × Challenges	Pre-2000: Subjectivity, variability, stress; Post-2010: Technical glitches, cheating	Challenges evolve with technology integration
8	Year × Requirements/Needs	Pre-2000: Standardization, training; Post-2010: Security, user-friendly tech	Needs adapt to emerging technological demands
9	Year × Solutions	Pre-2000: Consensus/multiple examiners; Post-2010: AI/proctoring	More technology-driven solutions
10	Year × Outcomes/Key Findings	Pre-2000: Qualitative positive; Post-2010: Statistical (p-values, correlations)	Increasingly rigorous, quantitative results
11	Year × Study Limitations	Pre-2000: Lack of data/generalizability; Post-2010: Tech-specific/small sample	Limitations reflect changing study designs
12	Country × Study Design	US: Retrospective/cohort; Canada: Observational; India: Mixed/narrative; Saudi: Comparative	Design differences tied to regional resources
13	Country × Target Population	US: Postgrad; India: Undergrad; Brazil: Elderly	Educational focus varies culturally
14	Country × Educational Setting	US: Multi/university; India: Department-specific; Brazil: Community	Setting shaped by healthcare/education systems
15	Country × Type of Exam	US: Mock/structured; India: Traditional/viva; Canada: Structured	US favors simulation; India retains traditional viva
16	Country × Technological Component	US: Virtual/video; India: Apps; Brazil: None	Technological component use highest in US, lowest in Brazil
17	Country × Challenges	US: Bias/reliability; India: Subjectivity/time; Brazil: Reproducibility	Challenges mirror local infrastructure
18	Country × Requirements/Needs	US: High-fidelity/feedback; India: Low-cost/standardization; Brazil: Simple tools	Needs based on resource context
19	Country × Solutions	US: Recording/simulation; India: Sensitization/apps; Brazil: Training	Solutions adapted to cultural context
20	Country × Outcomes/Key F indings	US: High pass rates/stats; India: Correlations/satisfaction; Brazil: Kappa/validity	Quantitative rigor more common in US
21	Country × Study Limitations	US: Single site/small sample; India: Scalability; Brazil: Recruitment	Limitations shaped by local capacity
22	Study Design × Target Population	Observational for postgrad; Comparative for undergrad	Design aligned to learner level
23	Study Design × Educational Setting	Quasi-experimental: University; Cross-sectional: Community	Design reflects setting characteristics
24	Study Design × Type of Exam	Quasi-experimental: Structured; Narrative: Traditional	Interventional designs introduce innovations
25	Study Design × Technological Component	Prospective: Virtual; Descriptive: None	Advanced designs adopt technology
26	Study Design × Challenges	Reliability analysis variability; Comparative bias	Design targets specific challenges
27	Study Design × Requirements/Needs	Observational: Training; Mixed: Security	Needs arise from study design
28	Study Design × Solutions	Quasi-experimental: Implementation; Qualitative: Refinement	Interventional designs test solutions
29	Study Design × Outcomes/Key Findings	Comparative: Correlation stats; Qualitative: Positive feedback	Comparative designs yield quantitative outputs
30	Study Design × Study Limitations	Descriptive: No stats; Prospective: Small sample	Limitations inherent to design type
31	Target Population × Educational Setting	Undergrad: University; Postgrad: Multi-site	Settings scale with learner level
32	Target Population × Type of Exam	Undergrad: Viva/group; Postgrad: Mock/structured	Complexity increases with learner advancement
33	Target Population × Technological Component	Students: None/video; Residents: Virtual/apps	Tech use rises with advanced training
34	Target Population × Challenges	Students: Stress/bias; Residents: Competency/reliability	Challenges vary by experience level
35	Target Population × Requirements/Needs	Students: Fairness/structure; Residents: Fidelity/training	Needs escalate with learner level
36	Target Population × Solutions	Students: Group/checklists; Residents: Simulation/recording	Solutions tailored to population
37	Target Population × Outcomes/Key Findings	Students: Satisfaction; Residents: Pass/reliability	Outcomes match educational goals
38	Target Population × Study Limitations	Students: Generalizability; Residents: Small sample	Common small n limitation
39	Educational Setting × Type of Exam	University: Structured; Community: Assessment	Format dictated by setting
40	Educational Setting × Technological Component	University: Video/apps; Home: None	Tech more common in formal institutions
41	Educational Setting × Challenges	University: Variability; Community: Reproducibility	Context-specific challenges
42	Educational Setting × Requirements/Needs	University: Standardization; Community: Simple tools	Needs tied to available resources
43	Educational Setting × Solutions	University: Consensus; Community: Training	Adapted solutions by context
44	Educational Setting × Outcomes/Key Findings	University: Reliability; Community: Validity	Different metrics emphasized
45	Educational Setting × Study Limitations	Single institution: Generalizability issues	Typical for university settings
46	Type of Exam × Technological Component	Structured: None/video; Traditional: None; Virtual: Apps	Tech enhances structured formats
47	Type of Exam × Challenges	Traditional: Subjectivity; Structured: Time/variability; Virtual: Glitches/bias	Format influences challenge types
48	Type of Exam × Requirements/Needs	Traditional: Standardization; Structured: Checklists/training; Virtual: Security/fidelity	Needs linked directly to format
49	Type of Exam × Solutions	Traditional: Feedback/training; Structured: Rubrics/multiple examiners; Virtual: AI/proctoring	Solutions mitigate format-specific issues
50	Type of Exam × Outcomes/Key Findings	Traditional: Qualitative; Structured: High reliability/stats; Virtual: Comparable performance	Structured/virtual better quantified
51	Type of Exam × Study Limitations	Traditional: Subjectivity; Structured: Small sample; Virtual: Tech bias	Limitations align with challenges
52	Technological Component × Challenges	Present: Glitches/compatibility/bias; Absent: Subjectivity/variability	Tech introduces new challenge types
53	Technological Component × Requirements/Needs	Present: Security/proctoring/user-friendly; Absent: Standardization/training	Needs shift with tech adoption
54	Technological Component × Solutions	Present: AI/voice modification; Absent: Checklists/consensus	Tech enables advanced solutions
55	Technological Component × Outcomes/Key Findings	Present: High satisfaction/comparable stats; Absent: Qualitative positive	Tech linked to better measurable outcomes
56	Technological Component × Study Limitations	Present: Compatibility/small sample; Absent: No stats/generalizability	Tech brings specific limitations
57	Challenges × Requirements/Needs	Variability/bias → Training/standardization/checklists	Direct mapping of challenges to needs
58	Challenges × Solutions	Subjectivity/variability → Rubrics/multiple examiners/tech	Solutions directly target challenges
59	Challenges × Outcomes/Key Findings	Addressed → Positive/reliable; Unaddressed → Inconsistent	Resolution improves outcomes
60	Challenges × Study Limitations	Persistent variability → Lack of stats/small sample	Unresolved challenges seen in limitations
61	Requirements/Needs × Solutions	Training/standardization → Implementation of training/rubrics	Needs lead to targeted solutions
62	Requirements/Needs × Outcomes/Key F indings	Met → High validity/reliability	Fulfilled needs yield positive results
63	Requirements/Needs × Study Limitations	Unmet → Inconsistency	Gaps reflected in limitations
64	Solutions × Outcomes/Key Findings	Structure/training/tech → Improved stats/satisfaction	Effective solutions yield strong outcomes
65	Solutions × Study Limitations	Structured formats (rubrics/checklists), examiner training, and tech adaptations (video/apps/virtual) → enhanced reliability, validity, satisfaction, and performance metrics	Targeted solutions directly correlate with measurable improvements in assessment quality and stakeholder perceptions, demonstrating efficacy in addressing challenges
66	Outcomes/Key Findings × Limitations	Positive outcomes often with small sample/no prospective design	Success tempered by methodological limits

### Comprehensive framework for optimized oral examinations

**Fig 5:**
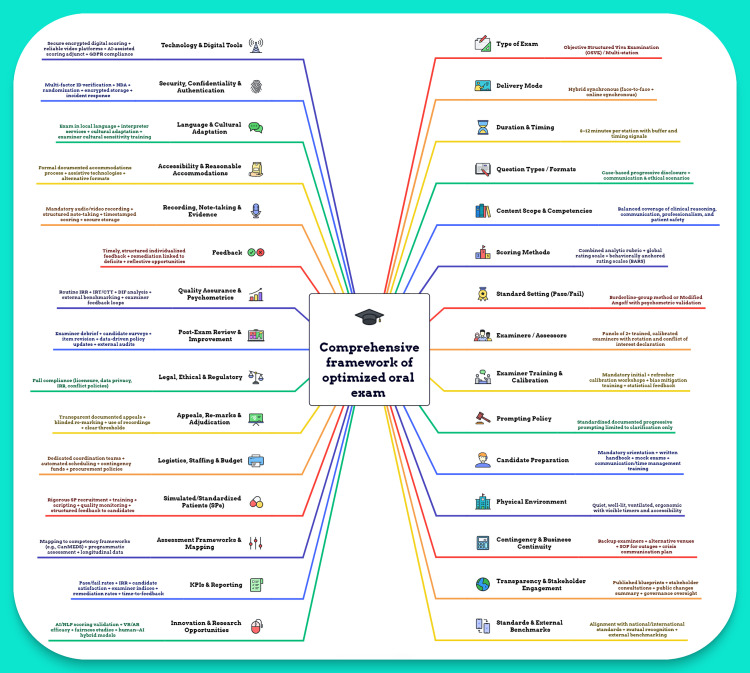
Comprehensive framework of optimized oral exam execution in medical and paramedical education.

Following a comprehensive analysis of all extracted data from the included studies, a unified framework for the implementation of oral examinations in medical and paramedical education was developed. Adherence to this framework is expected to optimize the outcomes of these assessments, enhancing their effectiveness and reliability in educational settings. Table 6 and Fig 5 illustrate this framework in detail.

**Table 6:** Comprehensive framework of optimized oral exam execution in medical and paramedical education.

Row	Dimension	Items/ Options	Best Evidence-Based Choice
1	Type of Exam	Traditional viva (Unstructured), Structured Oral Examination (SOE), Objective Structured Viva Examination (OSVE)/ Multi-station, Case-based viva (Single-case or multi-case), Problem-based oral, Competency-based oral (Based on competency frameworks), Entrustable Professional Activities (EPAs) oral, Portfolio-based viva (Portfolio defense), Oral component of OSCE/ integrated oral station, Thesis/dissertation defense (PhD, MSc), High-stakes licensing oral (board/registration), Peer-assessment viva, Peer-assisted/ near-peer viva, Group oral exam/ viva-voce, Panel/jury oral (Multi-disciplinary examiners), Simulated patient–led oral (Standardized or real patient present), Research viva vs clinical viva (Purpose distinction), Mixed-format (e.g., partly SOE + partly OSVE), Asynchronous recorded oral (non-interactive – candidate records), Live-streamed oral for remote panels, VR/Metaverse-based oral (Interactive virtual scenario), AR-assisted viva (Augmented reality – added visual content), Telemedicine-style oral (Connected to patient file or remote patient contact)	Objective Structured Viva Examination (OSVE)/ Multi-station
2	Delivery Mode	Face-to-face (In-person), Fully online synchronous (Zoom, Teams, WebEx), Hybrid (some stations in-person some online), Asynchronous online (Recorded responses reviewed later), Remote-proctored online (remote invigilation), Synchronous remote with local proctor, Mobile-app based oral (candidate answers via app), VR/AR platforms (Oculus, Haptic labs), Telepresence robots (Examiner represented by robot in room), Embedded bedside viva (At patient bedside), Simulation-lab integrated (Mannequin or interactive scenario), Field-based viva (Workplace/community based), Hybrid asynchronous/synchronous (e.g., theory recorded, practical live)	Hybrid synchronous (face-to-face + online synchronous)
3	Duration & Timing	Per candidate: 5–10/ 10–15/ 15–30/ 30–45/ 45–90 minutes, Per station: 4–8/ 8–12/ 12–20 minutes, Longer sessions for defenses: 2–6 hours or multiple consecutive days, Break times for candidates and examiners, Buffer times for technical delays or contingencies, Slotting algorithm (first-come, randomized, stratified), Warning signals (1 min, 30 sec left), Time for immediate feedback (in formative exams)	8–12 minutes per station with buffer and timing signals
4	Question Types/ Formats	Open-ended, Closed/short-answer factual, Case-based (single or progressive unfolding), Multi-step progressive disclosure, Interpretation of images/radiology/pathology/videos, Data analysis (lab results, ABG, ECG interpretation), Ethical dilemmas/ professionalism scenarios, Communication scenarios/ role-play with SP, Procedural walkthrough/ stepwise demonstration, Differential diagnosis generation, Management planning (short- and long-term), Evidence appraisal/ critical appraisal, Reflective questioning/ practice prompts, Team-based/ interprofessional scenarios, Situational judgment tests (oral SJT), OSCE-integrated stations with viva, Objective structured clinical judgment (OSCJ), Prompted viva (guided with cues), Open-book viva (allowed resources), Closed-book viva, Time-limited micro-viva (very short targeted),	Case-based progressive disclosure + communication & ethical scenarios
5	Content Scope & Competencies	Basic sciences knowledge, Clinical knowledge, Clinical reasoning, Management decision-making, Patient-centered communication skills, Professionalism and ethics, Procedural knowledge & technical skills, Interprofessional collaboration skills, Evidence-based practice (EBM), Research skills & article critique, Patient safety & risk management, Community-based health care, Legal & regulatory knowledge, Management skills & health systems, Cultural & intercultural sensitivity in clinical care	Balanced coverage of clinical reasoning, communication, professionalism, and patient safety
6	Scoring Methods	Ranking/ relative ordering, Numeric scoring/ points, Global rating scale (1–7), Checklist (done/not done), Analytic rubric (criteria-based), Combined analytic + global, Entrustment scales (1–5), Pass/fail binary, Weighted scoring (domain weighting), Anchoring vignettes for examiner calibration, AI-assisted automated scoring suggestions, NLP-based scoring of recorded answers (experimental), Safety deduction penalties, Behaviorally anchored rating scales (BARS), Checklist + narrative comments (quant + qual), Consensus scoring (post-discussion), Score aggregation rules (mean, median, trimmed mean), Tie-breaking rules (e.g., global rating priority)	Combined analytic rubric + global rating scale + behaviorally anchored rating scales (BARS)
7	Standard Setting (Pass/Fail)	Fixed cut-score (e.g., 50%), Angoff method, Modified Angoff, Borderline-group/ borderline regression, Hofstee compromise, Contrasting groups method, Bookmark method (for categorized items), CTT/IRT informed (psychometric) standard setting, Multifaceted standard setting (combining methods), Programmatic standard setting (integrated with other data)	Borderline-group method or Modified Angoff with psychometric validation
8	Examiners/ Assessors	Number: single examiner, two independent examiners, panel 3+, jury board. Specialty: specialist, interdisciplinary, generalist. Experience: experienced, novice, trained. Training status: calibrated, untrained. Duties: question asking, scoring, independent monitoring. Rotation: station rotation or fixed. Involvement of SPs/trainees/students in scoring, Quality observer presence, External examiner presence, Remote external examiners (via streaming), Examiner workload limits (max sessions/day), Conflict of interest declaration, Evaluator certification programs, Examiner remuneration/ honoraria, Examiner performance tracking (consistency, severity), Peer review of examiners (cross-check)	Panels of 2+ trained, calibrated examiners with rotation and conflict of interest declaration
9	Examiner Training & Calibration	Written guidelines, Calibration workshops (using recorded samples), Frame-of-reference training, Bias-awareness and mitigation training, Rater drift monitoring and re-calibration, Anchor videos/ exemplar performances, Double-marking pilot sessions, Statistical feedback to examiners (inter-rater reliability reports), Mandatory refresher courses, Certification & recertification intervals, Online modules for remote examiners, Competency in giving feedback training, Legal/ethical responsibilities briefing	Mandatory initial + refresher calibration workshops + bias mitigation training + statistical feedback
10	Prompting Policy	No prompting allowed, Clarification-only (limited ambiguity removal), Standardized follow-up questions (pre-approved), Progressive prompting (neutral to leading; documented), Allowed cues for communication-impaired candidates, Scripted probe questions (identical for all), Candidate-initiated clarification policy	Standardized documented progressive prompting limited to clarification only
11	Candidate Preparation	Orientation session (live or recorded), Written handbook with format, rubric, rules, Exemplar recorded orals, Mandatory mock viva/ formative practice, Simulation center exposure (for case-based), Time management training, Cultural/communication workshops (for language learners), Accessibility accommodations orientation, Consent for recording & data use, Instructions on permitted materials (open/closed book)	Mandatory orientation + written handbook + mock exams + communication/time management training
12	Physical Environment	Quiet and confidential room, Proper ventilation, lighting, temperature control, Ergonomic chairs, Audio settings (microphone, acoustics), AV equipment (cameras, monitors, speakers), Digital clocks/timers visible, Waiting area for candidates, Accessibility for disabled persons (ramps, elevators), Hygiene and emergency facilities, High-speed internet and secure network, Technical room for online support, Safety stations (handwashing, PPE), Physical distancing to reduce noise	Quiet, well-lit, ventilated, ergonomic with visible timers and accessibility
13	Technology & Digital Tools	Digital scoring system (tablet/web app), Secure audio/video recording, Video conferencing platforms (Zoom, Teams, BigBlueButton), Proctoring solutions (live, AI automated, hybrid), Biometric authentication (fingerprint, face), Secure exam browser (lockdown), VR simulation/ haptic devices, AI-assisted scoring suggestions/ NLP analysis, Speech-to-text transcription, Automated timestamping & audit logs, Cloud storage with encryption/ on-premise servers, LMS interoperability (Moodle, Canvas), Analytics dashboards (real-time scoring, rater stats), Automated notifications & scheduling, Backup power (UPS, generators), Data retention & deletion automation, Digital consent forms & logging	Secure encrypted digital scoring + reliable video platforms + AI-assisted scoring adjunct + GDPR compliance
14	Security, Confidentiality & Authentication	Encrypted access to question bank, Randomization of questions/stations between candidates, Candidate identity verification (ID, biometrics), NDA for examiners & staff, Direct/electronic monitoring to prevent cheating, Chain-of-custody mechanisms for data/files, Physical security protocols (locked rooms, lobby cameras), Encryption of recordings and data, Compliance with privacy laws (GDPR, HIPAA), Recording usage, retention and deletion policies, Incident response plan for breaches, Logging & tamper-evident audit trails	Multi-factor ID verification + NDA + randomization + encrypted storage + incident response
15	Language & Cultural Adaptation	Exam in local/ official language, Exam in foreign language (for migrants/international), Translation/ back-translation of questions, Culturally adapted vignettes, Interpreter provision (simultaneous), Bilingual examiners or stations, Language-appropriate scoring rubrics, Accommodations for non-native speakers (extra time, clarifications), Cultural sensitivity training for exam writers/examiners	Exam in local language + interpreter services + cultural adaptation + examiner cultural sensitivity training
16	Accessibility & Reasonable Accommodations	Extra time, additional breaks, Written versions of questions (for deaf), Sign language interpreters, Text-to-speech or speech-to-text software, Accessible physical environment (wheelchair), Audio/lighting adjustments (sensory), Alternative assessment formats if oral not feasible, Documented accommodation request & approval	Formal documented accommodations process + assistive technologies + alternative formats
17	Recording, Note-taking & Evidence	Formal audio/video recording, Structured note-taking forms, Transcription (human or automated), Timestamped scoring entries, Saving narrative comments with scores, Storage of raw data for audits/appeals, Redaction protocol for sensitive data, Metadata collection (examiner ID, time, location)	Mandatory audio/video recording + structured note-taking + timestamped scoring + secure storage
18	Feedback	Immediate oral feedback (formative), Structured written feedback (summative/formative), Video-review sessions with examiner, Individualized performance report, Remediation plans linked to deficits, Opportunities for reflective practice, Peer feedback sessions (formative), Feedback timing policy (within defined days), Feedback appeals/ review process, Anonymized cohort feedback (aggregate)	Timely, structured individualized feedback + remediation linked to deficits + reflective opportunities
19	Quality Assurance & Psychometrics	Inter-rater reliability analysis (IRR, ICC, kappa), Classical Test Theory (item difficulty, discrimination), Item Response Theory (IRT)/Rasch modeling, Generalizability Theory (sources of error), Reliability estimates by station/type/time, Standard error of measurement (SEM), Discrimination indices, Differential item functioning (DIF) for bias, Equating multiple forms, Sampling adequacy for psychometrics, Pre/post pilot item analysis, External benchmarking with other institutions, Examiner monitoring and feedback loops	Routine IRR + IRT/CTT + DIF analysis + external benchmarking + examiner feedback loops
20	Post-Exam Review & Improvement	Examiner debrief meetings, Candidate surveys (experience, fairness), Review & revision of problematic items, Archiving & version control of question bank, Policy updates based on incidents/analytics, External audit/accreditation reporting, Longitudinal cohort performance tracking, Remediation program updates, Publishing anonymized psychometric reports, Change control for assessment blueprints	Examiner debrief + candidate surveys + item revision + data-driven policy updates + external audits
21	Legal, Ethical & Regulatory	Compliance with national licensure/board, Data protection & privacy law compliance, Consent for recording and data use, Informed consent for SP involvement, Institutional Review Board (IRB)/ethics oversight, Malpractice/liability coverage, Examiner conflict of interest policy, Transparency and due process for appeals, Anti-discrimination policies and legal recourse, Record retention policies by law, Equal opportunity for all candidates	Full compliance (licensure, data privacy, IRB, conflict policies)
22	Appeals, Re-marks & Adjudication	Documented appeals process & timelines, Re-mark policies (who re-marks, blinded?), Arbitration/ appeals committee composition, Use of recordings in appeals, Thresholds for automatic review, Outcomes of appeal (grade change, re-exam), Remediation vs repeat exam policies, Reporting to accreditation bodies if breaches occur	Transparent documented appeals + blinded re-marking + use of recordings + clear thresholds
23	Logistics, Staffing & Budget	Staffing (coordinators, proctors, IT, SP coordinators), Budget for venue, AV, examiner fees, SPs, tech licenses, Scheduling systems (automated, conflict mgmt), Transport & accommodation budget, Contingency funds for delays/failures, Inventory of physical props & equipment, Procurement policies for tech vendors, Insurance costs (liability, equipment)	Dedicated coordination teams + automated scheduling + contingency funds + procurement policies
24	Simulated/Standardized Patients (SPs)	Recruitment strategies (actors, trained patients), SP training & scripting consistency, SP checklists and scoring (patient perspective), SP compensation & scheduling, Quality monitoring of SP performance, SP safety and wellbeing policies, SP feedback to candidates (structured), SP authenticity measures (standardization checks), Simulated family members/ interprofessional SP scenarios	Rigorous SP recruitment + training + scripting + quality monitoring + structured feedback to candidates
25	Assessment Frameworks & Mapping	Mapping to competency frameworks (CanMEDS, ACGME, GMC), Programmatic assessment approach (multiple low-stakes data), Longitudinal assessment strategies, Entrustability/EPAs mapping, Learning outcomes alignment & blueprint traceability, Curricular mapping to program outcomes & accreditation, Multi-source data triangulation (mini-CEX, OSCE, workplace-based),	Mapping to competency frameworks (e.g., CanMEDS) + programmatic assessment + longitudinal data
26	KPIs & Reporting	Pass rate, fail rate, borderline rate, Inter-rater reliability (ICC), Average station difficulty, Candidate satisfaction scores, Examiner reliability/leniency indices, Remediation completion rates, Time-to-feedback metrics, Cost per candidate, Audit logs and incident reports, Yearly QA reports for governance	Pass/fail rates + IRR + candidate satisfaction + examiner indices + remediation rates + time-to-feedback
27	Innovation & Research Opportunities	AI/NLP scoring validation, VR/AR efficacy studies, Feasibility of asynchronous oral grading, Fairness studies across demographics, Process mining of candidate-examiner interactions, Ethical research on proctoring tech impact, Advances in haptics for procedural viva, Human–AI hybrid assessment models	AI/NLP scoring validation + VR/AR efficacy + fairness studies + human–AI hybrid models
28	Contingency & Business Continuity	Backup examiners (on-call), Backup venues/ remote fallback setup, Alternative question sets if leakage suspected, SOP for power/network outage, Crisis communication plan, Illness response protocols (COVID etc.), Disaster recovery of digital records, Mental-health incident protocol (candidate distress)	Backup examiners + alternative venues + SOP for outages + crisis communication plan
29	Transparency & Stakeholder Engagement	Published exam blueprint & policies, Stakeholder consultations, Public summary of assessment changes, Feedback & complaint channels, External examiners involvement in policy, Governance committees and oversight	Published blueprints + stakeholder consultations + public changes summary + governance oversight
30	Standards & External Benchmarks	ISO/ educational standards applicability, Alignment with national accreditation standards, Comparability frameworks for international candidates, Mutual recognition agreements for licensure, External benchmarking against similar institutions	Alignment with national/international standards + mutual recognition + external benchmarking

## Discussion

This systematic review synthesizes evidence from 102 studies on the issues, requirements, and solutions for optimizing oral examinations in medical and paramedical education, highlighting a predominant focus on structured formats, persistent challenges such as examiner variability, and emerging technological integrations. The findings reveal that SOEs were the most common format, demonstrating superior reliability and validity compared to traditional unstructured viva voce, with improvements in inter-rater agreement post-standardization. These outcomes align with recent literature emphasizing the psychometric advantages of SOEs. For instance, a meta-analysis by Rahman et al. reported high validity and reliability for structured viva in health professions education, with overall acceptability rates of 79.8% among learners (p < 0.001), mirroring our observation of enhanced learner satisfaction in structured formats [14]. Similarly, a study by Pernar et al. on SOEs in undergraduate medical education found them effective in assessing clinical reasoning and providing real-time feedback, which corroborates our data on SOEs’ alignment with higher-order skills like decision-making and communication [15].

The geographical and temporal distribution of studies, with a surge in publications post-2000 and concentrations in North America and Europe, reflects evolving educational paradigms driven by competency-based frameworks such as CanMEDS and ACGME. This trend may explain the shift toward structured assessments, as global accreditation bodies increasingly demand evidence-based evaluation methods to ensure fairness and equity [16]. In comparison, a scoping review by Janke et al. on high-stakes examinations in higher education identified similar growth in oral assessment research, attributing it to the need for authentic evaluations that simulate clinical interactions, particularly in resource-constrained settings like those in Asia and Oceania represented in our review [17]. The predominance of medicine over paramedical fields like nursing in our included studies underscores a disciplinary bias, potentially due to the high-stakes nature of medical certification exams, where oral formats are integral for assessing procedural and ethical competencies. This is consistent with findings from a systematic review by Wang et al., which highlighted SOEs’ effectiveness in lab-based physiology sessions for medical students, improving performance metrics by addressing misconceptions through interactive probing [18].

Challenges identified in our review, such as examiner variability and student anxiety, persist despite interventions, likely attributable to inherent subjectivity in human-led assessments and the psychological pressures of high-stakes environments. These issues are exacerbated in traditional formats, where inter-rater reliability is lower, as evidenced by our data showing unstructured exams contributing to 27.5% of format-specific problems. Comparative studies support this: a randomized trial by Akkaraju demonstrated that oral exams, when unstructured, amplify bias and stress, reducing validity, whereas structured variants mitigate these through rubrics and calibration [19]. The post-COVID-19 emergence of virtual formats introduced new challenges like technical disruptions, but also solutions such as video recording for bias mitigation. This evolution is echoed in a narrative review by Khalaf et al. on online exams in dental education during the pandemic, which reported comparable outcomes to in-person assessments (p > 0.05) but highlighted connectivity issues as barriers, similar to our findings on equity concerns including language barriers and access disparities [20]. The limited technological integration in our review may stem from institutional inertia and resource limitations, particularly in low- and middle-income countries, where pre-pandemic reliance on face-to-face methods predominated. However, post-pandemic analyses, such as those by Hytönen et al., indicate accelerated adoption of digital tools like Zoom for OSCE-integrated orals, improving accessibility and reducing costs ($34.60 per resident in one study), which aligns with our framework’s recommendation for hybrid synchronous delivery [21].

Solutions proposed in our synthesis, including examiner training and standardization, were effective in enhancing reliability, likely because they address root causes like halo effects and rater drift through calibration and rubrics. This is substantiated by a quasi-experimental study by Lepp et al., which integrated oral exams across pharmacy courses, yielding higher satisfaction and performance due to structured feedback mechanisms [22]. The thematic requirements in Table 3, emphasizing curricular alignment and technological needs, explain improved outcomes in structured formats by ensuring validity through blueprinting and psychometric validation. For example, correlations between oral scores and OSCEs in our review indicate that orals assess unique non-cognitive domains, a finding replicated in a validity study by Pernar et al., where SOEs reduced racial grading disparities post-standardization [23]. The observed patterns in Table 5, such as the shift from traditional to virtual formats post-2010, can be interpreted as responses to global disruptions like COVID-19, fostering innovation in assessment to maintain educational continuity while enhancing equity [24].

Limitations prevalent in our included studies, including restricted generalizability and small sample sizes, mirror broader challenges in assessment research, where single-institution designs limit external validity. These align with critiques in a systematic review by Memon et al. on oral exams in postgraduate medical education, which noted biases from non-randomized methodologies [25]. Despite these, our comprehensive framework (Table 6) offers a blueprint for optimized execution, integrating evidence-based choices like multi-station OSVEs and AI-assisted scoring, which could mitigate biases and improve feasibility in diverse settings.

In terms of implications, our findings underscore the need to extend innovations beyond medicine into paramedical education, where oral examinations remain underutilized despite their potential to assess essential competencies. Targeted interventions should also address inequities in technological adoption across low- and middle-income countries. Future research must prioritize multicenter and longitudinal studies to evaluate the predictive validity of virtual oral exams for clinical performance, while exploring artificial intelligence and natural language processing to enhance objectivity and minimize bias [26]. By implementing these strategies, oral examinations can evolve into robust, equitable, and competency-driven tools that better prepare health professionals for real-world practice.

## Conclusion

This systematic review of 102 studies highlights that SOEs provide superior reliability, validity, fairness, and learner satisfaction compared to traditional viva formats. Key strategies for improvement include examiner training, question and scoring standardization, and the use of multiple examiners, all of which reduce subjectivity and enhance psychometric robustness. Technological tools such as video recording, virtual platforms, and digital scoring systems further support transparency, accessibility, and equity. Nevertheless, challenges persist, including examiner variability, student anxiety, logistical constraints, and equity-related issues such as language barriers and unequal access to technology. The underrepresentation of paramedical fields underscores the need for broader application and evaluation beyond medicine. Future research should focus on multicenter and longitudinal designs to strengthen generalizability and examine the predictive validity of oral exams for clinical performance. Emerging innovations—such as AI- and NLP-assisted scoring, VR/AR-enabled environments, and programmatic assessment frameworks—offer promising avenues for enhancing objectivity and scalability. In conclusion, oral examinations remain indispensable for assessing competencies such as clinical reasoning, ethical judgment, and communication. Through structured formats, examiner calibration, technological integration, and alignment with competency-based frameworks, oral exams can evolve into reliable, equitable, and learner-centered assessments that better prepare health professionals for real-world practice.

## Supporting information

S1 File Additional file 1. Details of the included studies.
(XLSX)

S2 File PRISMA check list. (DOCX)

## Abbreviations

PRISMA: preferred reporting items for systematic reviews and meta-analyses
QUADAS: quality assessment of diagnostic accuracy studies
SOE: structured oral examinations
OSCE: objective structured clinical examination
ICC: interclass correlation coefficient
OSVE: objective structured viva examination
AI: artificial intelligence
MBBS: bachelor of medicine, bachelor of surgery
PGY: post-graduate year
USA: United states of America
COVID-19: coronavirus disease-SARS-CoV-2
App: application
EPA: entrustable professional activities
PhD: doctor of philosophy
MSc: master of science
VR: virtual reality
AR: augmented reality
SJT: situational judgment tests
OSCJ: objective structured clinical judgment
EBM: evidence-based medicine
NLP: natural language processing
BARS: behaviorally anchored rating scales
CTT: classical test theory
IRT: item response theory
SP: standardized patient
AV: audiovisual
PPE: personal protective equipment
LMS: learning management systems
UPS: uninterruptible power supply
GDPR: general data protection regulation
HIPAA: health insurance portability and accountability act
IRR: inter-rater reliability
SEM: standard error of measurement
DIF: differential item functioning
IT: information technology
IRB: institutional review board
QA: quality assessment
CEX: clinical evaluation exercise
ACGME: Accreditation Council for Graduate Medical Education
CanMEDS: Canadian Medical Education Directives for Specialists
