# Supplementary material for: Structured approaches to improving outcomes of oral exam in medical and paramedical education: A systematic review and evidence-based framework
Source: PLoS One. 2026 Aug 10;21(8):e0355461. doi: 10.1371/journal.pone.0355461 (PMC13456202; doi:10.1371/journal.pone.0355461)
Supplement: Supplementary file 1 [file pone.0355461.s002.docx]

| **Section/Topic** | **Item #** | **Checklist Item** | **Reported?** | **Explanation and Location in Article** |
| --- | --- | --- | --- | --- |
| **Title** | 1 | Identify the report as a systematic review. | Yes | The title explicitly includes "A Systematic Review and Evidence-Based Framework." (Title section) |
| **Abstract** | 2 | See the PRISMA 2020 for Abstracts checklist. | Yes | The abstract is structured and covers background, methods (PRISMA, databases, inclusion/exclusion, quality appraisal), results (102 studies, key findings on challenges/solutions), and conclusions. It aligns with PRISMA Abstracts guidelines (e.g., objectives, eligibility, synthesis methods, limitations). (Abstract section) |
| **Introduction** | 3 | Describe the rationale for the review in the context of existing knowledge. | Yes | Rationale discusses advantages/disadvantages of oral exams, persistent challenges (reliability, bias), and gaps in synthesized evidence, citing prior literature [1-12]. (Background section) |
|  | 4 | Provide an explicit statement of the objective(s) or question(s) the review addresses. | Yes | PICO-framed question: "Among medical and paramedical students undergoing oral examinations (Population), what assessment strategies, requirements or interventions (Intervention), compared to traditional methods or no interventions (Comparison), are effective in improving oral examination outcomes (Outcome)?" Aims to synthesize issues, requirements, solutions. (Review Question subsection in Materials and Methods; also in Background) |
| **Methods** | 5 | Specify the inclusion and exclusion criteria for the review and how studies were grouped for the syntheses. | Yes | Inclusion: English-language full-text studies on oral assessments in medical/paramedical education. Exclusion: Editorials, commentaries, reviews, opinion pieces. No year limit. Studies grouped thematically (e.g., by challenges, solutions). (Inclusion and Exclusion Criteria subsection) |
|  | 6 | Specify all databases, registers, websites, organisations, reference lists and other sources searched or consulted to identify studies. Specify the date when each source was last searched or consulted. | Yes | Databases: PubMed, Web of Science, Scopus, CINAHL, ERIC, BIE, PsycINFO, IEEE, ProQuest, MEDLINE, Cochrane Library, Embase, SID, ISC (14 total), Google Scholar. Also: reference lists, handsearching, grey literature, email alerts. Last searched: June 23, 2025. (Sources of Information subsection) |
|  | 7 | Present the full search strategies for all databases, registers and websites, including any filters and limits used. | Yes | Full syntax provided in Table 1 (#1 AND #2 with keywords for oral exams and medical/paramedical fields). No filters/limits mentioned beyond English full-text. (Search Strategy subsection; Table 1) |
|  | 8 | Specify the methods used to decide whether a study met the inclusion criteria of the review, including how many reviewers screened each record and each report retrieved, whether they worked independently, and if applicable, details of automation tools used in the process. | Yes | Three independent reviewers (A.T.M., S.Gh., M.Gh.) screened titles/abstracts/full texts using a spreadsheet; exclusion by ≥2 votes. No automation mentioned. (Data Collection Procedure subsection) |
|  | 9 | Specify the methods used to collect data from reports, including how many reviewers collected data from each report, whether they worked independently, any processes for obtaining or confirming data from study investigators, and if applicable, details of automation tools used in the process. | Yes | Three reviewers extracted data independently using a uniform form (title, authors, year, etc.); consolidated and reviewed by independent investigator. No missing data. No investigator contact or automation. (Data Collection Procedure subsection) |
|  | 10a | List and define all outcomes for which data were sought. Specify whether all results that were compatible with each outcome domain in each study were sought, and if not, the methods used to decide which results to collect. | Yes | Outcomes: Study characteristics, challenges, requirements, solutions, outcomes/key findings (e.g., reliability, validity, pass rates). All compatible results sought; no selective collection mentioned. (Data Collection Procedure; Results subsections) |
|  | 10b | List and define all other variables for which data were sought (e.g. participant and intervention characteristics, funding sources). Describe any assumptions made about any missing or unclear information. | Yes | Variables: Country, design, population, setting, exam type, technology, limitations. No assumptions mentioned; no missing data reported. (Data Collection Procedure; Table 2-6) |
|  | 11 | Specify the methods used to assess risk of bias in the included studies, including details of the tool(s) used, how many reviewers assessed each study and whether they worked independently, and if applicable, details of automation tools used in the process. | Yes | QUADAS checklist (14 items; yes/no/unclear); two independent reviewers; high quality if ≥7 yes. Disagreements resolved by consensus. No automation. (Quality Evaluation subsection) |
|  | 12 | Specify for each outcome the effect measure(s) (e.g. risk ratio, mean difference) used in the synthesis or presentation of results. | Yes | No meta-analysis; qualitative synthesis. Measures: Cronbach's alpha, ICC, correlations (r), pass rates (%). Presented descriptively. (Results subsections on reliability/validity) |
|  | 13a | Describe the processes used to decide which studies were eligible for each synthesis. | Yes | All 102 eligible studies synthesized thematically; eligibility based on inclusion criteria and QUADAS. Tabulated characteristics compared to planned groups (e.g., by exam type). (Results; Table 5) |
|  | 13b | Describe any methods required to prepare the data for presentation or synthesis, such as handling of multi-arm trials, or multiple assessments of the same outcome. | Yes | Data consolidated in spreadsheet; thematic coding for challenges/solutions; no multi-arm handling mentioned (not applicable). (Quality Evaluation; Results) |
|  | 13c | Describe any methods used to tabulate or visually display results of individual studies and syntheses. | Yes | Tables (2-6), figures (1-5), word cloud (Figure 3), histogram/map (Figure 2), software: Excel, XMind, yED. (Results; Quality Evaluation) |
|  | 13d | Describe any methods used to synthesize results and provide a rationale for the choice(s). If meta-analysis was performed, describe the model(s), method(s) to identify the presence and extent of statistical heterogeneity, and software package(s) used. | Yes | Thematic analysis and matrix patterns (Table 5) for correlations; rationale: to identify patterns/challenges/solutions. No meta-analysis. (Methods; Results; Table 5) |
|  | 13e | Describe any methods used to explore possible causes of heterogeneity among study results (e.g. subgroup analysis, meta-regression). | Yes | Heterogeneity explored via thematic domains (e.g., by discipline, format); no formal subgroup/meta-regression. (Results subsections) |
|  | 13f | Describe any sensitivity analyses conducted to assess robustness of the synthesized results. | No | Not reported; no meta-analysis or sensitivity analyses. |
|  | 14 | Describe any methods used to assess risk of bias due to missing results in a synthesis (arising from reporting biases). | Yes | Grey literature searched to reduce publication bias; QUADAS for overall bias. (Sources of Information; Quality Evaluation) |
|  | 15 | Describe any methods used to assess certainty (or confidence) in the body of evidence for an outcome. | Yes | QUADAS for quality; limitations discussed (e.g., generalizability). No formal GRADE or certainty tool. (Quality Evaluation; Limitations in Results) |
| **Results** | 16a | Describe the results of the search and selection process, from the number of records identified in the search to the number of studies included in the review, ideally using a flow diagram. | Yes | 25,594 records → 102 included; detailed in text and PRISMA flow diagram (Figure 1). (Results) |
|  | 16b | Cite studies that might appear to meet the inclusion criteria, but which were excluded, and explain why they were excluded. | Yes | Exclusions: Non-English (3), not meeting criteria (72), no full text (4). No specific citations, but reasons provided. (Results) |
|  | 17 | Cite each included study and present its characteristics. | Yes | 102 studies summarized (e.g., by design, discipline); details in Additional File 1 (not provided here). No individual citations in main text. (Results; Table 5) |
|  | 18 | Present assessments of risk of bias for each included study. | Yes | QUADAS used; high/low quality classified, but not per-study results presented. (Quality Evaluation; Results) |
|  | 19 | For all outcomes, present, for each study: (a) summary statistics for each group (where appropriate) and (b) an effect estimate and its precision, ideally using structured tables or plots. | Yes | Aggregated statistics (e.g., median Cronbach's 0.75, ICC 0.47–0.82); no per-study estimates/plots. (Results subsections on reliability/outcomes) |
|  | 20a | For each synthesis, briefly summarise the characteristics and risk of bias among contributing studies. | Yes | Syntheses by themes (e.g., 102 studies, mostly high quality via QUADAS; bias in 59.2%). (Results; Table 4) |
|  | 20b | Present results of all statistical syntheses conducted. If meta-analysis was done, present for each the summary estimate and its precision and measures of statistical heterogeneity. If comparing groups, describe the direction of the effect. | Yes | No meta-analysis; descriptive stats (e.g., percentages, ranges) for syntheses. Directions: SOEs superior (e.g., higher reliability). (Results) |
|  | 20c | Present results of all investigations of possible causes of heterogeneity among study results. | Yes | Heterogeneity via patterns (Table 5); e.g., post-2000 surge, disciplinary bias. (Results; Table 5) |
|  | 20d | Present results of all sensitivity analyses conducted to assess the robustness of the synthesized results. | No | Not conducted/reported. |
|  | 21 | Present assessments of risk of bias due to missing results (arising from reporting biases) for each synthesis assessed. | Yes | Publication bias mitigated via grey literature; reporting bias in 59.2% studies. (Results; Table 4) |
|  | 22 | Present assessments of certainty (or confidence) in the body of evidence for each outcome assessed. | Yes | Evidence robust for SOEs but limited by generalizability/small samples (Table 4). No formal certainty grading. (Results; Discussion) |
| **Discussion** | 23a | Provide a general interpretation of the results in the context of other evidence. | Yes | SOEs superior; aligns with cited reviews/meta-analyses (e.g., Rahman et al. [14]). (Discussion) |
|  | 23b | Discuss any limitations of the evidence included in the review. | Yes | Generalizability (91.3%), small samples (49.5%), biases (Table 4). (Results; Discussion) |
|  | 23c | Discuss any limitations of the review processes used. | Yes | No pre-registration; potential bias in search/selection. (Search Strategy; Discussion) |
|  | 23d | Discuss implications of the results for practice, policy, and future research. | Yes | Framework for optimization (Table 6); need multicenter/longitudinal studies, AI innovations. (Discussion; Conclusion) |
| **Other Information** | 24a | Provide registration information for the review, including register name and registration number, or state that the review was not registered. | Yes | Not registered. (Search Strategy) |
|  | 24b | Indicate where the review protocol can be accessed, or state that a protocol was not prepared. | Yes | Not prepared/pre-registered. (Search Strategy) |
|  | 24c | Describe and explain any amendments to information provided at registration or in the protocol. | N/A | No registration/protocol. |
|  | 25 | Describe sources of financial or non-financial support for the review, and the role of the funders or sponsors in the review. | Yes | Funded by Medical Education Research Center, Tabriz University of Medical Sciences (grant 77220); role not specified. (Funding; Acknowledgements) |
|  | 26 | Declare any competing interests of review authors. | Yes | None declared. (Competing interests) |
|  | 27 | Report which of the following are publicly available and where they can be found: template data collection forms; data extracted from included studies; data used for all analyses; analytic code; any other materials used in the review. | Yes | Dataset in article and Additional File 1; extraction form described. (Availability of data and materials) |
