# Supplementary material for: Structured approaches to improving outcomes of oral exam in medical and paramedical education: A systematic review and evidence-based framework
Source: PLoS One. 2026 Aug 10;21(8):e0355461. doi: 10.1371/journal.pone.0355461 (PMC13456202; doi:10.1371/journal.pone.0355461)
Supplement: Supplementary file 2 [file pone.0355461.s004.docx]

**Reviewer #1:**

Comment: I do have one very minor question - the authors mention investigating unpublished reports. What were these and how did they access them if they were not published?

Response: Thank you very much for this valuable comment. We agree that the description of the grey literature search was insufficiently detailed in the original manuscript. To clarify this issue, we have revised the "Sources of Information" section to explicitly describe the types of grey literature searched. The corresponding text has been added to the Methods section.

**Sources of Information**

A thorough literature search was performed across multiple electronic databases up to June 23, 2025. These databases included PubMed, Web of Science, Scopus, CINAHL, ERIC, BIE, PsycINFO, IEEE, ProQuest, MEDLINE, Cochrane Library, Embase, SID, and ISC. In addition to peer-reviewed publications, we searched grey literature sources, including conference proceedings, books, dissertations, university repositories, institutional reports, and Google Scholar to reduce publication bias. These sources were identified through database searches, manual reference screening, and institutional repositories. Although grey literature was screened, only studies meeting all predefined eligibility criteria were included in the final review. To maintain awareness of new research, email alerts were configured in the databases to notify the authors of new studies matching the inclusion criteria, based on saved search queries as of June 23, 2025.

**additional requirements:**

1. Please ensure that your manuscript meets PLOS ONE's style requirements, including those for file naming.

Response: The manuscript has been revised to fully comply with PLOS ONE formatting and style requirements, including file naming and template guidelines.

2. Your ethics statement should only appear in the Methods section of your manuscript. If your ethics statement is written in any section besides the Methods, please delete it from any other section.

Response: The ethics statement has been moved and is now included only in the Methods section of the manuscript.

3. We note that Figure 2 in your submission contain map images which may be copyrighted. All PLOS content is published under the Creative Commons Attribution License (CC BY 4.0), which means that the manuscript, images, and Supporting Information files will be freely available online, and any third party is permitted to access, download, copy, distribute, and use these materials in any way, even commercially, with proper attribution. For these reasons, we cannot publish previously copyrighted maps or satellite images created using proprietary data, such as Google software (Google Maps, Street View, and Earth).

Response: Regarding Fig 2, the comments have been carefully addressed. The world map shown in Fig 2 was created by the authors using a public-domain base map (Natural Earth). We acknowledge the concern regarding copyrighted map sources and confirm that all necessary corrections have been made accordingly. This issue has been resolved and implemented in the revised manuscript.
