# Supplementary material for: Structured approaches to improving outcomes of oral exam in medical and paramedical education: A systematic review and evidence-based framework
Source: PLoS One. 2026 Aug 10;21(8):e0355461. doi: 10.1371/journal.pone.0355461 (PMC13456202; doi:10.1371/journal.pone.0355461)
Supplement: Peer review history

# Peer review: Decision Letter 0

**DOI:** 10.1371/journal.pone.0355461.r001

**Authors:** Santhi Silambanan

25 Jun 2026

Dear Dr. Ghaffarifar,

Thank you for submitting your manuscript to PLOS ONE. After careful consideration, we feel that it has merit but does not fully meet PLOS ONE’s publication criteria as it currently stands. Therefore, we invite you to submit a revised version of the manuscript that addresses the points raised during the review process.

Please submit your revised manuscript by Jul 25 2026 11:59PM. If you will need more time than this to complete your revisions, please reply to this message or contact the journal office at plosone@plos.org. When you're ready to submit your revision, log on to https://www.editorialmanager.com/pone/ and select the 'Submissions Needing Revision' folder to locate your manuscript file.

- A letter that responds to each point raised by the academic editor and reviewer(s). You should upload this letter as a separate file labeled 'Response to Reviewers'.
- A marked-up copy of your manuscript that highlights changes made to the original version. You should upload this as a separate file labeled 'Revised Manuscript with Track Changes'.
- An unmarked version of your revised paper without tracked changes. You should upload this as a separate file labeled 'Manuscript'.

If you would like to make changes to your financial disclosure, please include your updated statement in your cover letter. Guidelines for resubmitting your figure files are available below the reviewer comments at the end of this letter.

If applicable, we recommend that you deposit your laboratory protocols in protocols.io to enhance the reproducibility of your results. Protocols.io assigns your protocol its own identifier (DOI) so that it can be cited independently in the future. For instructions see: https://journals.plos.org/plosone/s/submission-guidelines#loc-laboratory-protocols. Additionally, PLOS ONE offers an option for publishing peer-reviewed Lab Protocol articles, which describe protocols hosted on protocols.io. Read more information on sharing protocols at https://plos.org/protocols?utm_medium=editorial-email&utm_source=authorletters&utm_campaign=protocols.

As the corresponding author, your ORCID iD is verified in the submission system and will appear in the published article. PLOS supports the use of ORCID, and we encourage all coauthors to register for an ORCID iD and use it as well. Please encourage your coauthors to verify their ORCID iD within the submission system before final acceptance, as unverified ORCID iDs will not appear in the published article. *Only*  the individual author can complete the verification step; PLOS staff *cannot*  verify ORCID iDs on behalf of authors.

We look forward to receiving your revised manuscript.

Kind regards,

Santhi Silambanan, MD, DNB

Academic Editor

PLOS One

Journal Requirements:

If the reviewer comments include a recommendation to cite specific previously published works, please review and evaluate these publications to determine whether they are relevant and should be cited. There is no requirement to cite these works unless the editor has indicated otherwise.

Please review your reference list to ensure that it is complete and correct. If you have cited papers that have been retracted, please include the rationale for doing so in the manuscript text, or remove these references and replace them with relevant current references. Any changes to the reference list should be mentioned in the rebuttal letter that accompanies your revised manuscript. If you need to cite a retracted article, indicate the article’s retracted status in the References list and also include a citation and full reference for the retraction notice.

Additional Editor Comments:

I request the authors to respond to one query raised by reviewer 1.

[Note: HTML markup is below. Please do not edit.]

Reviewers' comments:

Reviewer's Responses to Questions

**Comments to the Author**

1. Is the manuscript technically sound, and do the data support the conclusions?

Reviewer #1: Yes

Reviewer #2: Yes

**********

2. Has the statistical analysis been performed appropriately and rigorously?

Reviewer #1: Yes

Reviewer #2: Yes

**********

3. Have the authors made all data underlying the findings in their manuscript fully available?

The PLOS Data policy

Reviewer #1: Yes

Reviewer #2: Yes

**********

4. Is the manuscript presented in an intelligible fashion and written in standard English?

Reviewer #1: Yes

Reviewer #2: Yes

**********

Reviewer #1: The authors have provided a comprehensive review of the types of oral examinations being carried out in a number of different situations and their perceived issues relating to reliability etc. They have reviewed a considerable amount of relevant data and provided 'Structured Approaches to Improving Outcomes in oral examinations' with an excellent set of 'Best evidence-based' choices for performing such oral examinations, which can facilitate further discussions in institutions where this type of examination is routinely used.

Helpful figures and supplementary materials have been included.

The review is well written with suitable explanations of acronyms and sources. All credit to the authors as it would seem that English is a second language.

I do have one very minor question - the authors mention investigating unpublished reports. What were these and how did they access them if they were not published?

Reviewer #2: The review article on Oral Examination in Medical and Paramedical Education is well written and provides a comprehensive overview of most of the important aspects observed in oral examinations. The manuscript is organized logically, and the discussion is clear and easy to follow.

As a review article, it successfully covers the major strengths, challenges, and practical considerations associated with oral assessment.

**********

what does this mean?). If published, this will include your full peer review and any attached files.

If you choose “no”, your identity will remain anonymous but your review may still be made public.

**Do you want your identity to be public for this peer review?**  For information about this choice, including consent withdrawal, please see our Privacy Policy

Reviewer #1: No

Reviewer #2: No

**********

[NOTE: If reviewer comments were submitted as an attachment file, they will be attached to this email and accessible via the submission site. Please log into your account, locate the manuscript record, and check for the action link "View Attachments". If this link does not appear, there are no attachment files.]

To ensure your figures meet our technical requirements, please review our figure guidelines: https://journals.plos.org/plosone/s/figures

You may also use PLOS’s free figure tool, NAAS, to help you prepare publication quality figures: https://journals.plos.org/plosone/s/figures#loc-tools-for-figure-preparation.

NAAS will assess whether your figures meet our technical requirements by comparing each figure against our figure specifications.

# Author comment: Author response to Decision Letter 1

**DOI:** 10.1371/journal.pone.0355461.r002

3 Jul 2026

Dear Editor-in-Chief,

We would like to express our sincere gratitude for the thoughtful and constructive comments provided during the review of our manuscript submitted to PLOS ONE. We have carefully considered all the reviewers’ suggestions and have addressed each point in detail in the revised version of the manuscript.

Reviewer #1:

Comment: I do have one very minor question - the authors mention investigating unpublished reports. What were these and how did they access them if they were not published?

Response: Thank you very much for this valuable comment. We agree that the description of the grey literature search was insufficiently detailed in the original manuscript. To clarify this issue, we have revised the "Sources of Information" section to explicitly describe the types of grey literature searched. The corresponding text has been added to the Methods section.

Sources of Information

A thorough literature search was performed across multiple electronic databases up to June 23, 2025. These databases included PubMed, Web of Science, Scopus, CINAHL, ERIC, BIE, PsycINFO, IEEE, ProQuest, MEDLINE, Cochrane Library, Embase, SID, and ISC. In addition to peer-reviewed publications, we searched grey literature sources, including conference proceedings, books, dissertations, university repositories, institutional reports, and Google Scholar to reduce publication bias. These sources were identified through database searches, manual reference screening, and institutional repositories. Although grey literature was screened, only studies meeting all predefined eligibility criteria were included in the final review. To maintain awareness of new research, email alerts were configured in the databases to notify the authors of new studies matching the inclusion criteria, based on saved search queries as of June 23, 2025.

additional requirements:

1. Please ensure that your manuscript meets PLOS ONE's style requirements, including those for file naming.

Response: The manuscript has been revised to fully comply with PLOS ONE formatting and style requirements, including file naming and template guidelines.

2. Your ethics statement should only appear in the Methods section of your manuscript. If your ethics statement is written in any section besides the Methods, please delete it from any other section.

Response: The ethics statement has been moved and is now included only in the Methods section of the manuscript.

3. We note that Figure 2 in your submission contain map images which may be copyrighted. All PLOS content is published under the Creative Commons Attribution License (CC BY 4.0), which means that the manuscript, images, and Supporting Information files will be freely available online, and any third party is permitted to access, download, copy, distribute, and use these materials in any way, even commercially, with proper attribution. For these reasons, we cannot publish previously copyrighted maps or satellite images created using proprietary data, such as Google software (Google Maps, Street View, and Earth).

Response: Regarding Fig 2, the comments have been carefully addressed. The world map shown in Fig 2 was created by the authors using a public-domain base map (Natural Earth). We acknowledge the concern regarding copyrighted map sources and confirm that all necessary corrections have been made accordingly. This issue has been resolved and implemented in the revised manuscript.

Attachment Submitted filename: Response to Reviewers.docx

# Editor report: Decision Letter 1

**DOI:** 10.1371/journal.pone.0355461.r003

**Authors:** Santhi Silambanan

22 Jul 2026

Structured Approaches to Improving Outcomes of Oral Exam in Medical and Paramedical Education: A Systematic Review and Evidence-Based Framework

PONE-D-26-23522R1

Dear Dr. Ghaffarifar,

We’re pleased to inform you that your manuscript has been judged scientifically suitable for publication and will be formally accepted for publication once it meets all outstanding technical requirements.

Within one week, you’ll receive an e-mail detailing the required amendments. When these have been addressed, you’ll receive a formal acceptance letter and your manuscript will be scheduled for publication.

An invoice will be generated when your article is formally accepted. Please note, if your institution has a publishing partnership with PLOS and your article meets the relevant criteria, all or part of your publication costs will be covered. Please make sure your user information is up-to-date by logging into Editorial Manager at Editorial Manager® and clicking the ‘Update My Information' link at the top of the page. For questions related to billing, please contact billing support.

If your institution or institutions have a press office, please notify them about your upcoming paper to help maximize its impact. If they’ll be preparing press materials, please inform our press team as soon as possible -- no later than 48 hours after receiving the formal acceptance. Your manuscript will remain under strict press embargo until 2 pm Eastern Time on the date of publication. For more information, please contact onepress@plos.org.

Kind regards,

Santhi Silambanan, MD, DNB

Academic Editor

PLOS One

# Editor report: Acceptance letter

**DOI:** 10.1371/journal.pone.0355461.r004

**Authors:** Santhi Silambanan

PONE-D-26-23522R1

PLOS One

Dear Dr. Ghaffarifar,

I'm pleased to inform you that your manuscript has been deemed suitable for publication in PLOS One. Congratulations! Your manuscript is now being handed over to our production team.

At this stage, our production department will prepare your paper for publication. This includes ensuring the following:

* All references, tables, and figures are properly cited

* All relevant supporting information is included in the manuscript submission,

* There are no issues that prevent the paper from being properly typeset

You will receive further instructions from the production team, including instructions on how to review your proof when it is ready. Please keep in mind that we are working through a large volume of accepted articles, so please give us a few days to review your paper and let you know the next and final steps.

Lastly, if your institution or institutions have a press office, please let them know about your upcoming paper now to help maximize its impact. If they'll be preparing press materials, please inform our press team within the next 48 hours. Your manuscript will remain under strict press embargo until 2 pm Eastern Time on the date of publication. For more information, please contact onepress@plos.org.

You will receive an invoice from PLOS for your publication fee after your manuscript has reached the completed accept phase. If you receive an email requesting payment before acceptance or for any other service, this may be a phishing scheme. Learn how to identify phishing emails and protect your accounts at https://explore.plos.org/phishing.

If we can help with anything else, please email us at customercare@plos.org.

Thank you for submitting your work to PLOS ONE and supporting open access.

Kind regards,

PLOS ONE Editorial Office Staff

on behalf of

Dr. Santhi Silambanan

Academic Editor

PLOS One
